# High quality health systems in the SDG era: Country-specific priorities for improving quality of care

**DOI:** 10.1371/journal.pmed.1002946

**Published:** 2019-10-03

**Authors:** Gagan Thapa, Manoj Jhalani, Sebastián García-Saisó, Address Malata, Sanam Roder-DeWan, Hannah H. Leslie

**Affiliations:** 1 Federal Parliament of Nepal, Kathmandu, Nepal; 2 National Health Mission, Ministry of Health and Family Welfare, New Delhi, India; 3 General Directorate for Quality of Healthcare and Education, Ministry of Health, Ciudad de México, Mexico; 4 Malawi University of Science and Technology, Limbe, Malawi; 5 Health Section, UNICEF, Dar es Salaam, Tanzania; 6 Health Systems: Impact Evaluation and Policy, Ifakara Health Institute, Dar es Salaam, Tanzania; 7 Department of Global Health and Population, Harvard T. H. Chan School of Public Health, Boston, Massachusetts, United States of America

## Abstract

Hannah Leslie and co-authors discuss priorities in individual countries for health system reform.

Long a concern in high-income countries, health system quality emerged as a truly global priority in 2018. Three major reports [1–3] and an increasing body of empirical work (e.g., [4–6]) identified deep and pervasive deficits in quality undermining progress toward the Sustainable Development Goal (SDG) of health and wellness for all by 2030. Whether this attention translates into effective action in the near future will depend on national leaders seizing the opportunity to change health systems for improved outcomes. How can policymakers and researchers coordinate to ensure that action is informed by evidence and that policy changes are assessed for generalizable insight? To address this question, we gathered leaders in global health active along a spectrum from generating insight in individual studies to making policy that affects millions of individuals and present some of their thoughts here. All have identified health system quality as a key element of progress; each brings a distinct perspective on what change will require in their country or context. As those setting the agenda in countries from India to Mexico, how do they see the future for health system quality? What is needed from the research community to accelerate progress towards improved health?

From Nepal, Gagan Thapa argues that strong political commitment is required to ensure that each individual has access to affordable and quality healthcare. Political actors need to be informed about issues related to affordability and other quality issues related to health services and have the political will to resolve them. Critical reform areas such as medical education, procurement of medicines, minimum quality standards, regulation, and partnerships with the private sector will require meaningful engagement of lawmakers as well as their commitment to increases in national budgets for health.

People in all countries must be empowered to demand health services that are responsive to their health needs. We have to be better at giving people platforms to express their health needs by including citizen voices during policymaking, budgeting, and planning processes of governments. Yet people are ill-informed about their rights to access quality and affordable healthcare. All too often, people do not access care because it is too expensive, inconvenient, remote from their household, or insensitive to their cultural practices. Many face catastrophic expenditures because of the high costs of surgery, medicines, and related costs for travel and accommodation. We need to have the political courage to include citizen voices in government processes, understand and document the difficulties people face, and use creative methods to resolve them.

Health has been for too long the sole domain of Ministries of Health and their supporting technical partners: WHO, UNICEF, and others. The language of health policy is far too complex, and its approaches are technical. Thus, political leaders have been prevented from taking up this worthy cause. As the High-Quality Health Systems in the SDG Era report points out [1], the quality agenda requires strong political commitment, and the language used in the health discourse must be understandable and directed toward political actors—extending from issues that people face and challenges in delivering health services to potential solutions for reform.

For Address Malata, one of the most compelling ideas for improving healthcare quality is that tried and tested measures that have improved quality of care in high-income countries cannot just be adopted for the health sector in low- and middle-income countries (LMICs). Country- and in some cases site-specific measures and practices need to be researched, tried, and tested in target areas before being implemented. In particular, it is evident that many LMICs like Malawi are not making significant progress in maternal healthcare as indicators are still poor [7]. A rise in noncommunicable diseases has also strained the healthcare system [8]. Therefore, there is a need to have a more strategic conversation among researchers, policymakers, educators, and practitioners in countries.

So as to induce policymakers to act, several types of evidence will be needed. First, robust data on mortality (especially maternal and neonatal) related to poor quality of care. Second, a systematic assessment of what difference minimal investment in various measures of quality care can make in saving lives. Finally, evidence is required to indicate that some quality of care packages are not necessarily too expensive to be employed.

In Tanzania, efforts to improve high perinatal and maternal mortality rates have stagnated [9]. The bottleneck is not with typical quality improvement targets, such as provider knowledge; it is much deeper, within the structure of the health system, asserts Sanam Roder-DeWan. Women and newborns are dying because the system is not designed to maximize its own assets. The majority of women are delivering in facilities where even highly skilled providers could not deliver the quality of care that is needed to save lives: emergency cesarean sections are impossible and often require resources and expertise that is several hours travel away. The system was designed to place delivery services for “low risk” women close to home. This is laudable but hinges heavily on the assumption that we can accurately risk-stratify women. Unfortunately we cannot, because approximately 30% of low-risk pregnancies develop complications [10]. Such a high proportion is far too great for remote facilities with poor referral links to cope. The system must be redesigned to allow all women to deliver in, or very close to, a facility with comprehensive emergency obstetric and newborn care (CEmONC) services [1]. Facilities without CEmONC capacity should specialize in care that can be expertly delivered at that level, such as antenatal and postnatal care. Other components of redesign are transport, dignified maternity waiting options, and midwifery-led low-intervention units. Tanzania has taken the first important step of expanding CEmONC capacity, and committing to fully redesigning service delivery is the next big step to take.

Manoj Jhalani notes that in India, at Sub-Health Centres catering to population groups of about 5,000 people, Auxiliary Nurse Midwives with short-duration midwifery training are expected to provide reproductive, maternal, and child health services, including delivery care. This strategy was designed at a time when access to safe delivery was constrained by cultural and geographic barriers [11]. Expanding such access for delivery has not led to commensurate reductions in maternal and early newborn mortality. Conversely, care for chronic diseases, including ambulatory care, is currently available at higher level facilities, limiting geographic and financial access, and compromising early detection and follow-up for treatment adherence. As a result, the burden of noncommunicable diseases has continued to increase [12]. Redesigning service delivery, wherein delivery and newborn care is provided at higher level facilities to ensure quality and long-term ambulatory care for chronic diseases is provided at peripheral facilities, is therefore the most transformative idea for India.

The provision of comprehensive primary healthcare visualizing this shift to delivery of improved quality care is already national policy in India, in the form of the Ayushman Bharat Yojana health scheme, but requires the use of regularly updated data through dashboards to provide evidence of gains in conjunction with patient satisfaction surveys, so as to sustain and accelerate policy commitment.

As countries reach better levels of access to healthcare, quality becomes even more visible as a key element to bridge the gap towards universal health coverage and better results in health, argues Sebastián García-Saisó. Mexico has over the past two decades made important efforts towards universal healthcare but has struggled to improve health outcomes despite the investment made in health. This is one of the most compelling arguments towards quality improvement: no matter how much investment goes into health service provision, if quality is not at the center of all health interventions then improved results will not automatically follow.

As a fragmented federal health system with multiple institutions and levels interacting in terms of health insurance and service provision, Mexico has developed an important number of quality indicators based on episodic measurement of structural elements and user experiences. Despite this consideration, the system has yet to implement robust transverse information systems in order to systematically evaluate and monitor health outcomes, including the capacity to monitor individual health outcomes and follow patients throughout the system. This is particularly relevant at local levels and primary health service provision. Overall, there is an urgent need to improve local decision-making towards quality improvement, emphasizing the role of health and healthcare in equity, and the need for better transparency and accountability in resource allocation and utilization.

Although priorities vary by country, health systems around the world are struggling to deliver high-quality care in the face of the long-standing challenges of maternal and child health and infectious diseases, along with the growing epidemic of noncommunicable disease. We call for research to channel people’s voices, develop tools specifically for LMIC settings, test the reshaping of health systems to align care delivery with minimum quality standards, and learn from individual patient outcomes over time to inform system strengthening. Only then will health systems truly deliver for all.
